# Association of irreversible electroporation treatment with overall survival in Stage III pancreatic ductal adenocarcinoma: DIRECT registry results

**DOI:** 10.1093/oncolo/oyag416

**Published:** 2026-10-14

**Authors:** Robert C G Martin II, Rebekah Ruth White, Malcolm M Bilimoria, Govindarajan Narayanan, Michael D Kluger, David A Iannitti, Patricio M Polanco, Chet W Hammill, Sean P Cleary, Robert Evans Heithaus, Theodore Welling, Carlos H F Chan

**Affiliations:** Division of Surgical Oncology, University of Louisville School of Medicine, Louisville, KY 40202, United States; Moores Cancer Center, University of California, San Diego, La Jolla, CA 92101, United States; Northwest Community Healthcare, Arlington Heights, IL 61312, United States; Miami Cancer Institute, Baptist Health South Florida, Miami, FL 33101, United States; Herbert Irving Comprehensive Cancer Center, Columbia University, New York, NY 10001, United States; Atrium Health Carolinas Medical Center, Charlotte, NC 28201, United States; University of Texas Southwestern Medical Center, Dallas, TX 75201, United States; Siteman Cancer Center, Washington University School of Medicine in St. Louis, St. Louis, MO 63001, United States; Present address: The Oregon Clinic, Portland, OR 97209, United States; Mayo Clinic, Rochester, MN 55901, United States; Present address: University Health Network, University of Toronto, Toronto, Ontario M4B 1B3, Canada; University of South Florida, Tampa, FL 33601, United States; NYU Langone Health, New York, NY 10282, United States; UC San Diego Health, University of California, San Diego, CA 92101, United States; University of Iowa Health Care, Iowa City, IA 52240, United States

**Keywords:** gastrointestinal cancers/pancreatic cancer, chemotherapy/combination chemotherapy, surgical oncology, irreversible electroporation

## Abstract

**Background:**

Irreversible electroporation (IRE) is used in selected patients with Stage III pancreatic ductal adenocarcinoma (PDAC), but its association with survival relative to standard-of-care (SOC) therapy remains uncertain.

**Methods:**

This multicenter observational study included 137 patients from the DIRECT registry: 99 treated within an IRE-based strategy and 38 receiving SOC without IRE. Overall survival (OS) from diagnosis was evaluated using propensity-score overlap weighting as the principal adjusted analysis. Conventional inverse probability of treatment weighting (IPTW), propensity-score matching, 3- and 6-month landmark analyses, and a time-dependent Cox model treating IRE as a time-varying exposure were sensitivity analyses. Restricted mean survival time (RMST) at 24 months was estimated.

**Results:**

Median time from diagnosis to enrollment was 8 months (IQR, 6-10) with IRE and 4 months (IQR, 3-6) with SOC (*P* < .0001). Unadjusted median OS was 28 months (95% CI, 23-33) and 15 months (95% CI, 12-21), respectively. Overlap weighting was associated with lower mortality with IRE (HR, 0.28; 95% bootstrap CI, 0.12-0.48). Twenty-four-month RMST was 21.22 vs 16.24 months, an absolute difference of 4.99 months (95% bootstrap CI, 1.15-8.12). The time-dependent analysis attenuated but retained the association (HR, 0.59; 95% bootstrap CI, 0.37-0.94). Other sensitivity analyses were directionally consistent.

**Conclusions:**

An IRE-based treatment strategy was associated with longer OS than SOC without IRE. Attenuation in the time-dependent analysis indicates that treatment timing and selection contributed to the observed difference. Given the nonrandomized design and potential residual confounding, these findings demonstrate association rather than an independent causal effect of IRE.

Implications for practice For selected patients with Stage III PDAC who remain without progression following initial systemic therapy, incorporation of IRE into a multimodality treatment strategy may be associated with longer survival compared with SOC alone. This association persisted across multiple approaches addressing measured confounding and treatment timing, although it was attenuated when IRE was modeled as a time-dependent exposure. These observational findings should support prospective evaluation and multidisciplinary treatment consideration rather than be interpreted as evidence of an independent causal benefit of IRE.

## Introduction

Pancreatic ductal adenocarcinoma (PDAC) is the most common form of pancreatic cancer, the third leading cause of cancer-related death in the United States, with a 5-year survival rate of around 10%.^1^ At the time of diagnosis, only 15-20% of pancreatic cancers are considered resectable.^2^ Treatment is complex for patients with Stage 3 pancreatic cancer (Stage 3 PC) with systemic chemotherapy with or without radiation as the current standard of care (SOC). Recent evidence suggests 5-fluorouracil, leucovorin, irinotecan, and oxaliplatin (FOLFIRINOX) and combination therapy with gemcitabine and nab-paclitaxel have improved efficacy over gemcitabine alone and are considered the optimal first-line therapy.^3^

For patients with Stage 3 locally advanced unresectable disease, irreversible electroporation (IRE) is a non-thermal ablation method that was cleared by the United States Food and Drug Administration (FDA) in 2010 under 510(k) K102329 and uses high-voltage short electrical pulses to change the permeability of the cell membrane, leading to cell death.^4–6^ Due to its non-thermal mechanism of action, since 2012 IRE has become a potential treatment option for Stage 3 PC, as it allows for the ablation of tumors while preserving surrounding vital structures.^7^^,^^12^ There are 2 operative clinical uses of IRE for pancreatic cancer: IRE for the ablation of unresectable lesions (in-situ ablation) and margin accentuation when resection is feasible, but there are concerns for a positive margin at vital structures.^8^ Historically, unresectable Stage 3 PC had a low median survival of 9-16 months depending on the treatment used.^9^^,^^10^ Emerging evidence suggests IRE may improve overall survival (OS) in this patient population.^11–16^ A large, prospective registry study of 200 patients with locally advanced pancreatic cancer (LAPC) reported a median OS from diagnosis of 25 months.^17^ Furthermore, PANFIRE-2, a prospective, phase II trial, studied the safety and efficacy of percutaneous IRE for LAPC and reported a median OS from diagnosis of 17 months which exceeded the target median OS for patients receiving no induction chemotherapy or gemcitabine-based induction chemotherapy (12 months) and for those receiving induction FOLFIRINOX (15 months).^18^

As a United States FDA Investigational Device Exemption (IDE) study, DIRECT is a registry study aimed to evaluate the effectiveness and safety of SOC plus IRE with the NanoKnife System compared to SOC alone for the treatment of Stage 3 PDAC.

A previous safety publication from the DIRECT registry demonstrated that the use of IRE for curative intent tumor ablation in combination with induction chemotherapy has equivalent morbidity and 90-day mortality rates when compared to SOC chemotherapy alone.^19^ This paper reports the OS and progression-free survival (PFS) of these cohorts. The present analysis evaluates whether treatment within an IRE-based strategy was associated with overall survival among patients with Stage III PDAC receiving contemporary systemic therapy, while explicitly accounting for measured treatment-selection differences and differential treatment timing.

## Methods

### Study design and patient selection

The DIRECT Registry (Clinicaltrials.gov NCT03899649) study is a prospective, observational registry of patients in the United States from 18 clinical sites. This study was not randomized and not blinded. Enrollment began in 08/05/2019 and ended in 02/05/2024, and patients were followed through at least 24 months, death, or loss to follow-up. The study included patients with Stage 3 Pancreatic ductal adenocarcinoma (PDAC) undergoing IRE with the NanoKnife System in addition to SOC in accordance with each participating sites’ guidelines, as well as a comparator cohort of patients with Stage 3 PDAC who received at least 3 months of SOC but did not receive IRE. Patients treated with IRE were prospectively enrolled from sites that provide IRE as part of clinical care (IRE sites) while the comparator cohort was comprised of prospectively enrolled patients and contemporaneous retrospective data collected on patients at DIRECT sites undergoing SOC. For the retrospective SOC patients, “enrollment” was defined as the point at which patients became eligible for the study after 3 months of SOC therapy (ie, were re-staged as Stage 3 to confirm they were neither down staged nor progressed following treatment). The SOC cohort was enrolled from 26/09/2019 to 17/06/2024. All enrolled patients (IRE and SOC) finished the study, and there was no attrition. The overall flow of patients from the original registry enrollment target through accrual, eligibility assessment, treatment classification, and inclusion in the present comparative analysis is shown in Figure 1A. Because the timing of treatment differed between cohorts, particularly with respect to the interval from diagnosis to enrollment and receipt of IRE, the temporal relationship among diagnosis, induction therapy, registry enrollment, IRE treatment, and follow-up is illustrated in Figure 1B.

**Figure 1.**
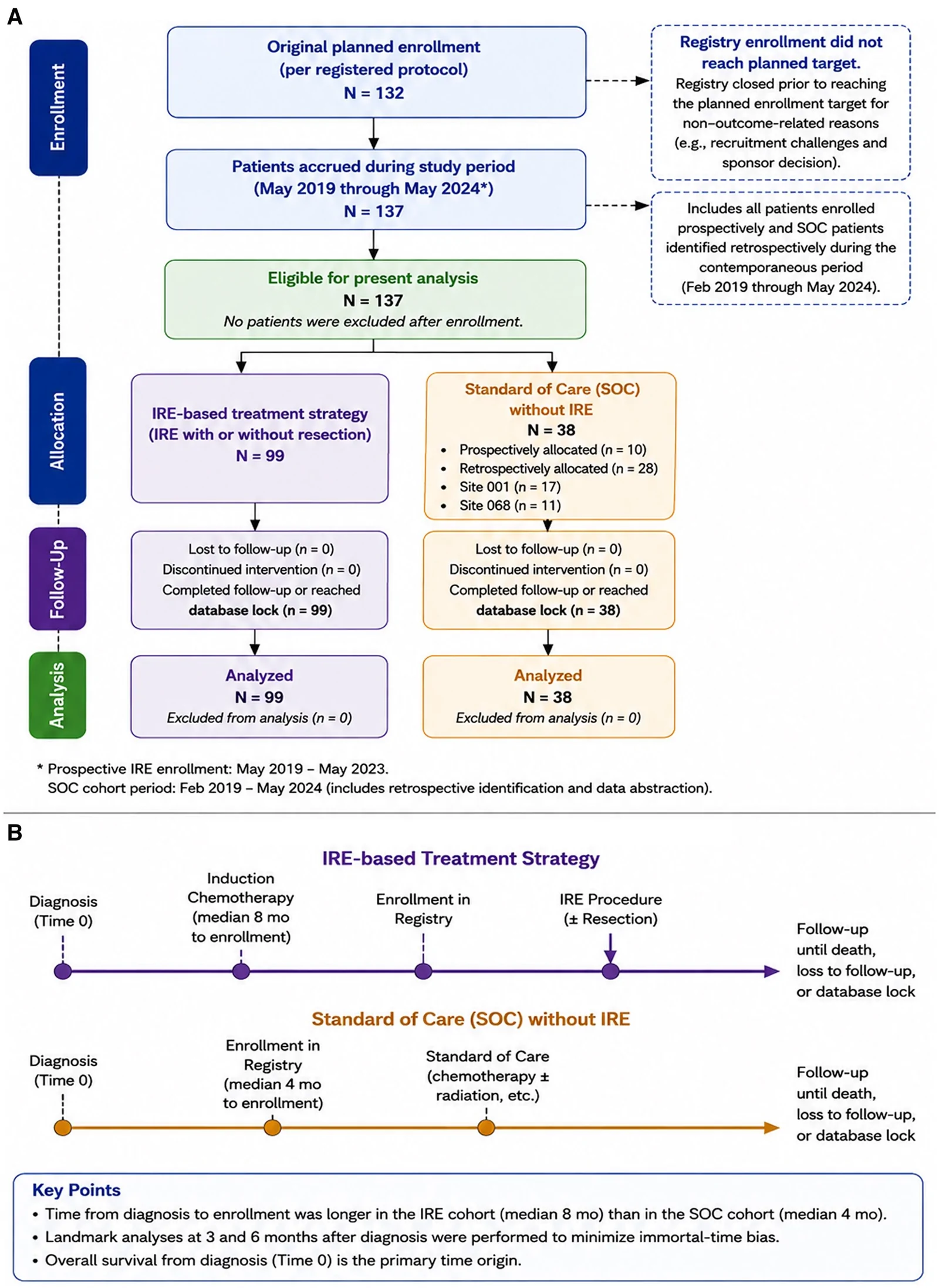
(A) Patient flow through the DIRECT Registry. The original registered protocol planned enrollment of 532 patients. The present analysis included all 137 eligible patients accrued during the study period, including 99 treated within an IRE-based treatment strategy and 38 receiving SOC without IRE. No eligible patient was excluded according to subsequent treatment response or survival outcome. IRE, irreversible electroporation; SOC, standard of care. (B) Study timeline and analytic time origin. Schematic representation of treatment and follow-up in the IRE-based and SOC treatment strategies. The figure illustrates the differential interval between diagnosis and registry enrollment and the additional interval between diagnosis and IRE treatment. These temporal differences motivated the use of OS from diagnosis as the primary time origin and the performance of landmark and time-dependent treatment analyses to address potential immortal-time and treatment selection bias.

### Registry enrollment and study status

The DIRECT registry was originally designed with a planned enrollment of 532 patients (266 patients per treatment cohort), as described in the registered study protocol (NCT03899649) (Figure S1). The planned enrollment represented the original recruitment target and was not achieved during the study enrollment period. Enrollment of patients undergoing IRE began in May 2019 and ended in May 2024, because of sponsor decision based on recruitment limitations and initial COVID-19 effects, which lead to a very slow initiation. The present analysis therefore includes all patients enrolled in the registry who satisfied the eligibility criteria for the comparative survival analysis and for whom the required outcome data were available at database lock, rather than a selectively sampled subset of the originally planned population.

The final analysis population consisted of 137 patients, including 99 patients treated within an IRE-based treatment strategy and 38 patients receiving SOC without IRE. The difference between the originally planned sample size and the final analytic population reflects lower-than-planned registry accrual rather than post hoc selection of patients according to treatment response or survival outcome. No survival-based stopping rule or interim efficacy analysis was used to determine study closure. The present report represents the first comparative survival analysis of the DIRECT registry.

Patients in both cohorts were enrolled at the completion of 3 months of initial SOC treatment for stage 3 PDAC. Patients continued to receive SOC chemotherapy after completing the required 3 months of treatment as per the treating principal investigator and/or treating physician. Site enrollment and specific inclusion and exclusion are included in Supplementary Methods.

### Treatment timing and analytic time origin

Although all patients were required to complete a minimum of 3 months of SOC before study eligibility, the interval from diagnosis to registry enrollment and subsequent IRE treatment varied between patients. Therefore, diagnosis, completion of induction treatment, registry eligibility/enrollment, IRE treatment, and subsequent follow-up were treated as distinct temporal events. Figure 1B illustrates these treatment pathways and the potential interval during which patients subsequently treated with IRE had to remain alive and progression-free before receiving the intervention. To minimize bias arising from these differences, OS from diagnosis was selected as the principal survival time origin, supplemented by landmark and time-dependent treatment analyses.

### Study procedure

Patients in the IRE cohort were treated with IRE using the NanoKnife System (AngioDynamics, Inc., Latham, NY, USA). IRE protocol (Clinicaltrials.gov NCT03899649) and patient selection with the NanoKnife System has been previously described.^19^^,^^20^

### Endpoints

The primary effectiveness endpoint for the present comparative analysis was OS, defined as time in months from diagnosis to death from any cause. Patients alive at the end of follow-up were censored at the last date on which vital status was known. OS from diagnosis was selected as the primary time origin because the interval from diagnosis to registry enrollment differed substantially between treatment groups.

Secondary effectiveness endpoints included OS from study enrollment and progression-free survival (PFS), defined as time from enrollment to the date of first documented disease progression or death from any cause, whichever occurred first.

AEs are presented including relationship to treatments received and AEs CTCAE grade 3 or higher.

### Statistical analysis

Statistical analyses were designed to account for the observational nature of the DIRECT registry, nonrandom treatment allocation, differences in the timing of treatment eligibility, and potential baseline imbalances between patients treated within an irreversible electroporation (IRE)-based treatment strategy and those receiving standard of care (SOC) without IRE. Because treatment assignment was not randomized, effect estimates are interpreted as measures of association rather than causal treatment effects. Full statistical analysis is included in the Supplementary methods.

*Effect estimates are reported with 2-sided 95% CIs. Two*-sided *P* values <.05 were considered statistically significant; however, interpretation emphasized effect size, precision, covariate balance, and consistency across sensitivity analyses rather than *P* values alone. Analyses were performed using SAS 9.4.

### Role of the sponsor

The study sponsor (AngioDynamics, Inc.) provided financial and logistical support but had no role in data collection or data interpretation. The academic investigators had full access to the complete dataset and retained final responsibility for data interpretation and the decision to submit the manuscript for publication.

## Results

### Patient characteristics and procedure details

Of the 532 patients originally planned for enrollment, 137 eligible patients were accrued and available for the final comparative analysis during the study period (Figure 1A). The analytic cohort included 99 patients treated within an IRE-based treatment strategy and 38 patients receiving SOC without IRE. No patients meeting the prespecified eligibility criteria for this analysis were excluded on the basis of subsequent treatment response or survival outcome.

Within the IRE cohort, 87 patients received open IRE and 12 received percutaneous IRE. The median (IQR) duration of follow-up was 16 (8-27) months for the IRE cohort and 10 (7-16) months for the SOC cohort. The median (IQR) age was 65 (58-71) years in the IRE cohort and 67 (61-75) years in the SOC cohort. Median (IQR) time from diagnosis to enrollment was 8 (6-10) months for the IRE cohort compared with 4 (3-6) months in the SOC cohort (*P* < 0.0001). Regarding induction (first-line) chemotherapy, most patients in both cohorts received FOLFIRINOX; 51% (50/99) in the IRE arm and 58% (24/38) in the SOC arm, with a median (IQR) of 3 (1-8) cycles in the IRE cohort and 4 (4-7) cycles in the SOC cohort (*P* = .79). Patients in the IRE cohort were more likely to have had prior radiation therapy: 38% (38/99) for IRE vs 3% (1/38) for SOC, *P* < .0001. Patient and disease characteristics are presented in Table 1 and were similar between cohorts. The procedure details for the IRE cohort are presented in Table S1.

**Table 1.** Patient and disease characteristics before and after propensity-score overlap weighting.

Characteristic	IRE (*N* = 99)	SOC (*N* = 38)	Unweighted SMD	Overlap-weighted SMD
**Age, years**				
** Mean (SD)**	64 (9)	67 (10)	0.335	<0.001
** Median (Q1-Q3)**	65 (58-71)	67 (61-75)		
**Sex, *n* (%)**				
** Male**	43 (43%)	18 (47%)	0.079	<0.001
** Female**	56 (57%)	20 (53%)		
**Race, *n* (%)**				
** Asian**	0 (0%)	2 (5%)		
** Black or African American**	6 (6%)	4 (11%)		
** White**	92 (93%)	29 (76%)		
** Unknown**	1 (1%)	3 (8%)		
**Ethnicity, *n* (%)**				
** Hispanic or Latino**	14 (14%)	2 (5%)		
** Not Hispanic or Latino**	85 (86%)	33 (87%)		
** Unknown**	0 (0%)	3 (8%)		
**ECOG performance status, *n* (%)**				
** 0**	57 (58%)	21 (55%)		
** 1**	42 (42%)	17 (45%)	0.047	<0.001
**Year of initial pancreatic cancer diagnosis**				
** Median year**	2020	2021	0.567	<0.001
** 2016**	1 (1%)	0 (0%)		
** 2018**	4 (4%)	0 (0%)		
** 2019**	10 (10%)	5 (13%)		
** 2020**	35 (35%)	5 (13%)		
** 2021**	35 (35%)	15 (40%)		
** 2022**	14 (14%)	10 (26%)		
** 2023**	0 (0%)	2 (5%)		
** 2024**	0 (0%)	1 (3%)		
**Tumor location, *n* (%)**				
** Head-involving**	58 (59%)	20 (53%)	0.120	<0.001
** Body/neck only**	38 (38%)	17 (45%)		
** Body/neck; tail**	3 (3%)	1 (3%)		
**Anterior/posterior tumor diameter, cm**				
** *n***	68	28		
** Mean (SD)**	2 (1)	3 (1)	0.634	<0.001
** Median (Q1-Q3)**	2 (2-3)	3 (2-4)		
**Axial tumor diameter, cm**				
** *n***	68	28		
** Mean (SD)**	2 (1)	3 (1)		
** Median (Q1-Q3)**	2 (2-3)	3 (2-4)		
**Cranial-caudal tumor diameter, cm**				
** *n***	62	28		
** Mean (SD)**	2 (1)	3 (1)		
** Median (Q1-Q3)**	3 (2-3)	3 (3-4)		
**Time from diagnosis to IRE, months**				
** *n***	99	N/A		
** Mean (SD)**	9 (5)	N/A		
** Median (Q1-Q3)**	8 (7-11)	N/A		
**Time from diagnosis to enrollment, months**				
** *n***	99	38		
** Mean (SD)**	9 (5)	5 (2)		
** Median (Q1-Q3)**	8 (6-10)	4 (3-6)		
**Chemotherapy prior to enrollment, *n* (%)**				
** FOLFIRINOX-containing**	73 (74%)	24 (63%)	0.229	<0.001
** Gemcitabine/nab-paclitaxel-containing**	37 (37%)	13 (34%)	0.256	<0.001
**Number of chemotherapy cycles prior to enrollment**				
** *n***	85	37		
** Mean (SD)**	5 (5)	5 (3)	0.009	<0.001
** Median (Q1-Q3)**	3 (1-8)	4 (4-7)		
**Prior radiation therapy, *n* (%)**				
** Yes**	38 (38%)	1 (3%)	0.988	<0.001
** No**	57 (58%)	35 (92%)		
** Unknown**	4 (4%)	2 (5%)		
**Baseline CA19-9, U/mL**				
** n**	89	38		
** Mean (SD)**	98 (168)	4023 (19526)		
** Median (Q1-Q3)**	30 (14-86)	67 (24-398)	0.637*	<0.001*

Values are presented as *n* (%), mean (SD), or median (Q1-Q3), as indicated. Standardized mean differences (SMDs) are reported as absolute values. SMDs are shown for covariates included in the propensity-score model; blank cells indicate descriptive variables or category levels not separately parameterized in the model. An absolute SMD <0.10 was prespecified as indicating adequate balance.

Abbreviations: ECOG = Eastern Cooperative Oncology Group; CA19-9 = carbohydrate antigen 19-9; IRE = irreversible electroporation; SOC = standard of care.

* CA19-9 SMD was calculated after log transformation. Overlap weighting achieved SMDs <0.001 for all modeled covariates.

### Post-enrollment chemotherapy and radiation therapy

There were similar dose density and duration of chemotherapy in both cohorts post-enrollment; 36% (36/99) patients in the IRE cohort and 74% (28/38) in the SOC cohort received a second line of chemotherapy with a similar median (IQR) number of cycles of chemotherapy between cohorts with 4 (3-7) for IRE cohort and 5 (4-8) for the SOC cohort (*P* = .29) (Table S3). Type of chemotherapy received was similar between cohorts with most patients receiving gemcitabine/nab-paclitaxel (41%, 26/137) or FOLFIRINOX (36%, 23/137). Overall, more SOC patients received third through- fifth lines of chemotherapy. Among patients with a known number of chemotherapy cycles, the median (IQR) cumulative number of cycles (first through third lines) was 6 (2-12) for the IRE cohort (*n* = 85) and 11 (7-13) for the SOC cohort (*n* = 37) (*P* = .01). Post-enrollment, 29% (29/99) of the IRE cohort received radiation and 40% (15/38) of the SOC cohort received radiation (*P* = .02). Combining pre- and post-enrollment therapy, 65% (64/99) of IRE patients received radiation, and 42% (16/38) of SOC patients received radiation (*P* = .004).

### Overall survival and adjusted comparative analyses

Overall survival from diagnosis was the primary survival endpoint. In the unweighted population, median OS from diagnosis was 28 months (95% CI, 23-33) for patients treated within an IRE-based treatment strategy and 15 months (95% CI, 12-21) for patients receiving SOC without IRE (log-rank *P* < .0001) (Table 2). The corresponding unadjusted Cox hazard ratio was 0.29 (95% CI, 0.19-0.45). These unadjusted estimates are presented descriptively and were not interpreted as causal treatment effects because treatment allocation and treatment timing differed between cohorts. Within the IRE cohort, OS was additionally calculated separately for patients receiving in-situ ablation (*n* = 62) and margin accentuation (*n* = 37) with a median OS of 18 months for the in-situ group (95% CI: 11-26) and 20 months for the margin accentuation group (95% CI: 14-28).

**Table 2.** Overall survival: descriptive, primary adjusted, and sensitivity analyses.

Analysis	Population/time origin	Effect estimate	95% CI/difference	Interpretive role
**Unadjusted median OS**	All patients; from diagnosis	IRE 28 mo vs SOC 15 mo	IRE 23-33; SOC 12-21	Descriptive
**Unadjusted Cox**	All patients; from diagnosis	HR 0.29	0.19-0.45	Descriptive
**Overlap-weighted Cox**	Propensity overlap population; from diagnosis	HR 0.28	Bootstrap CI 0.12-0.48	Principal adjusted analysis
**Overlap-weighted 24-mo RMST**	Propensity overlap population; from diagnosis	IRE 21.22 mo vs SOC 16.24 mo	Difference +4.99 mo (1.15-8.12)	Principal absolute effect measure
**Stabilized IPTW Cox**	All weighted patients; from diagnosis	HR 0.29	Bootstrap CI 0.14-0.55	Sensitivity; residual imbalance remained
**Propensity-score matched Cox**	29 matched pairs; from diagnosis	HR 0.35	0.19-0.63	Sensitivity
**3-month landmark**	Alive/progression-free at 3 mo	HR 0.29	Bootstrap CI 0.14-0.54	Sensitivity
**6-month landmark**	Alive/progression-free at 6 mo	HR 0.35	Bootstrap CI 0.15-0.65	Sensitivity
**Time-dependent IRE Cox**	IRE treated as time-varying exposure	HR 0.59	Bootstrap CI 0.37-0.94	Key immortal-time bias analysis

Overall survival outcomes from diagnosis across the unadjusted comparison, principal overlap-weighted analysis and prespecified sensitivity analyses.

Abbreviations: IRE = irreversible electroporation; SOC = standard of care; mo = months; OS = overall survival.

Propensity-score weighting materially changed the inferential framework without changing the direction of the observed association. Conventional IPTW produced an adjusted HR of 0.29 (95% bootstrap CI, 0.14-0.55), but several baseline covariates remained above the prespecified absolute SMD threshold of 0.10 after IPTW, indicating residual imbalance and limited common support (Figure 2).

**Figure 2.**
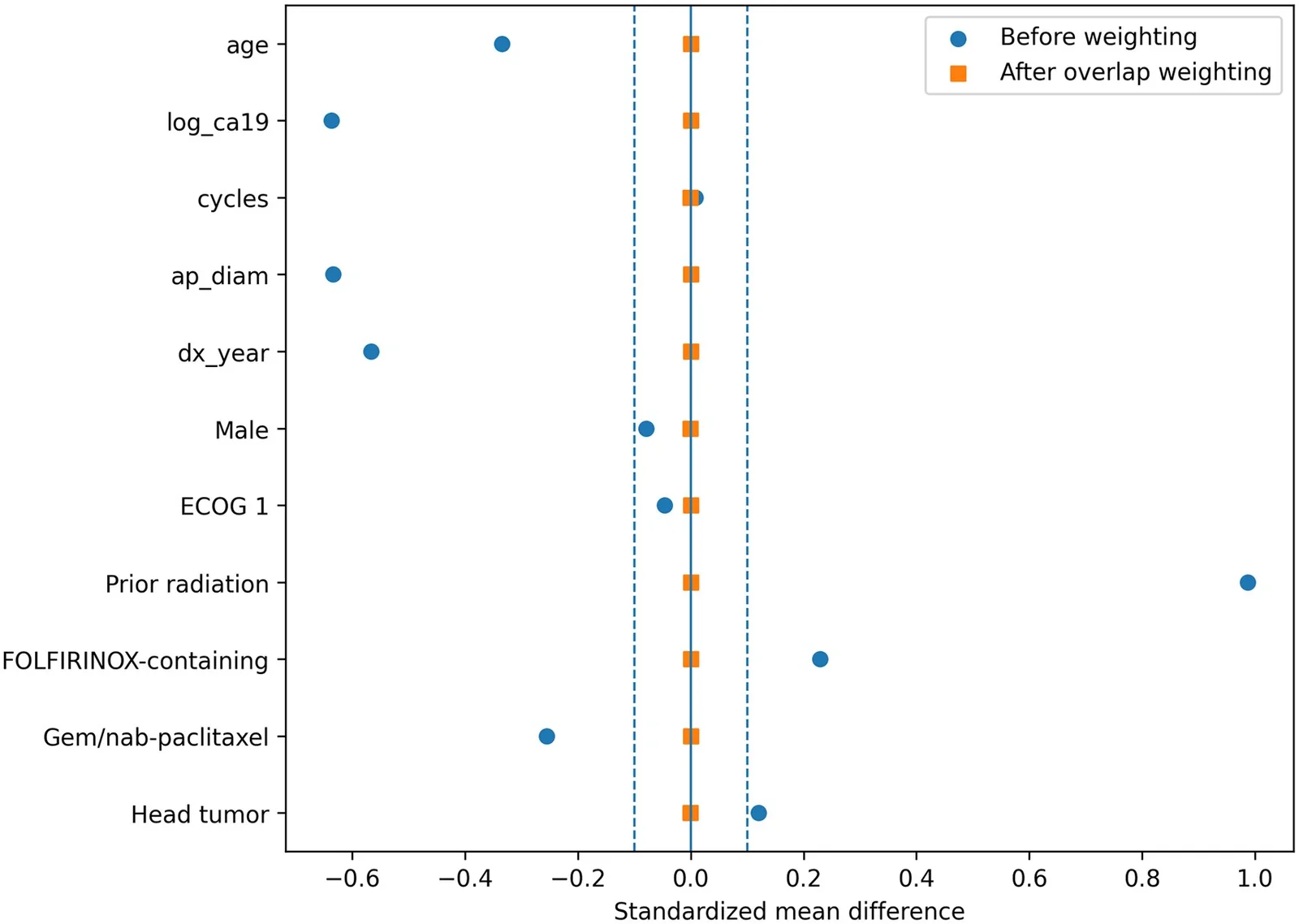
Covariate balance before and after inverse probability of treatment weighting. Love plot displaying absolute standardized mean differences (SMDs) for prespecified baseline covariates comparing patients treated within an irreversible electroporation (IRE)-based treatment strategy with those receiving standard of care (SOC) without IRE, before and after stabilized inverse probability of treatment weighting (IPTW). Dashed vertical reference lines indicate an absolute SMD of 0.10, the pre-specified threshold for acceptable covariate balance. Although IPTW improved balance for several measured covariates, residual imbalance remained for selected characteristics, supporting the use of overlap weighting as the principal adjusted observational analysis. SMD, standardized mean difference; IPTW, inverse probability of treatment weighting; IRE, irreversible electroporation; SOC, standard of care.

Overlap weighting achieved substantially better balance across the modeled baseline covariates and was therefore used as the principal adjusted observational analysis (Figure 2). Conventional IPTW left substantial residual imbalance for several variables (Figure 2). In the overlap-weighted population, the HR for mortality associated with the IRE-based treatment strategy was 0.28 (95% bootstrap CI, 0.12-0.48). At 24 months from diagnosis, overlap-weighted RMST was 21.22 months for IRE and 16.24 months for SOC, corresponding to an absolute difference of 4.99 months (95% bootstrap CI, 1.15-8.12).

The principal finding was directionally consistent across multiple sensitivity analyses. Propensity-score matching yielded 29 matched pairs and an HR of 0.35 (95% CI, 0.19-0.63).

The 3-month landmark analysis produced an HR of 0.29 (95% bootstrap CI, 0.14-0.54), while the 6-month landmark analysis produced an HR of 0.35 (95% bootstrap CI, 0.15-0.65) (Figure 3). Most importantly, in the time-dependent Cox analysis, patients were considered unexposed until the actual date of IRE treatment; the association with mortality was attenuated but remained present (HR, 0.59; 95% bootstrap CI, 0.37-0.94). This attenuation indicates that differential treatment timing contributed to the magnitude of the unadjusted association and provides a more conservative estimate after specifically addressing immortal-time bias. The *E*-value for the overlap-weighted point estimate was 6.68 and for the confidence-limit closest to the null was 3.63. The principal overlap-weighted estimate and results of the prespecified sensitivity analyses are summarized in Figure 4.

**Figure 3.**
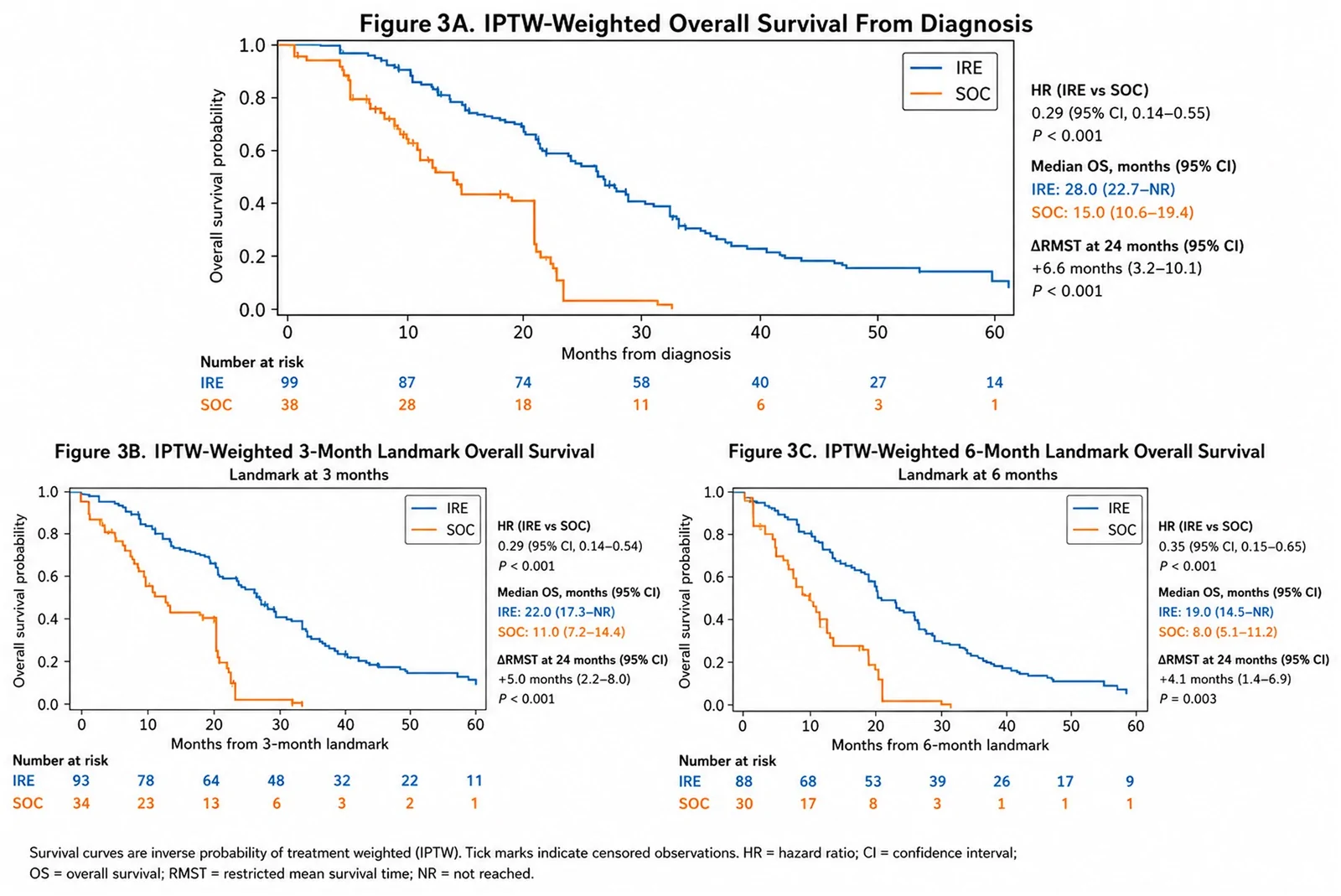
Overall survival from diagnosis and prespecified landmark analyses according to treatment strategy. Weighted Kaplan-Meier estimates of overall survival comparing patients treated within an irreversible electroporation (IRE)-based treatment strategy with patients receiving standard of care (SOC) without IRE. Panel (A) demonstrates overall survival from diagnosis in the propensity-score weighted population. Panel (B) demonstrates survival conditional on being alive and progression-free at the 3-month landmark, and Panel (C) demonstrates survival conditional on being alive and progression-free at the 6-month landmark. The 3-month landmark analysis yielded a hazard ratio (HR) for mortality of 0.29 (95% bootstrap CI, 0.14-0.54), and the 6-month landmark analysis yielded an HR of 0.35 (95% bootstrap CI, 0.15-0.65). Landmark analyses were performed as sensitivity analyses to evaluate the influence of differential treatment timing and patient selection after diagnosis. IRE, irreversible electroporation; SOC, standard of care; HR, hazard ratio.

**Figure 4.**
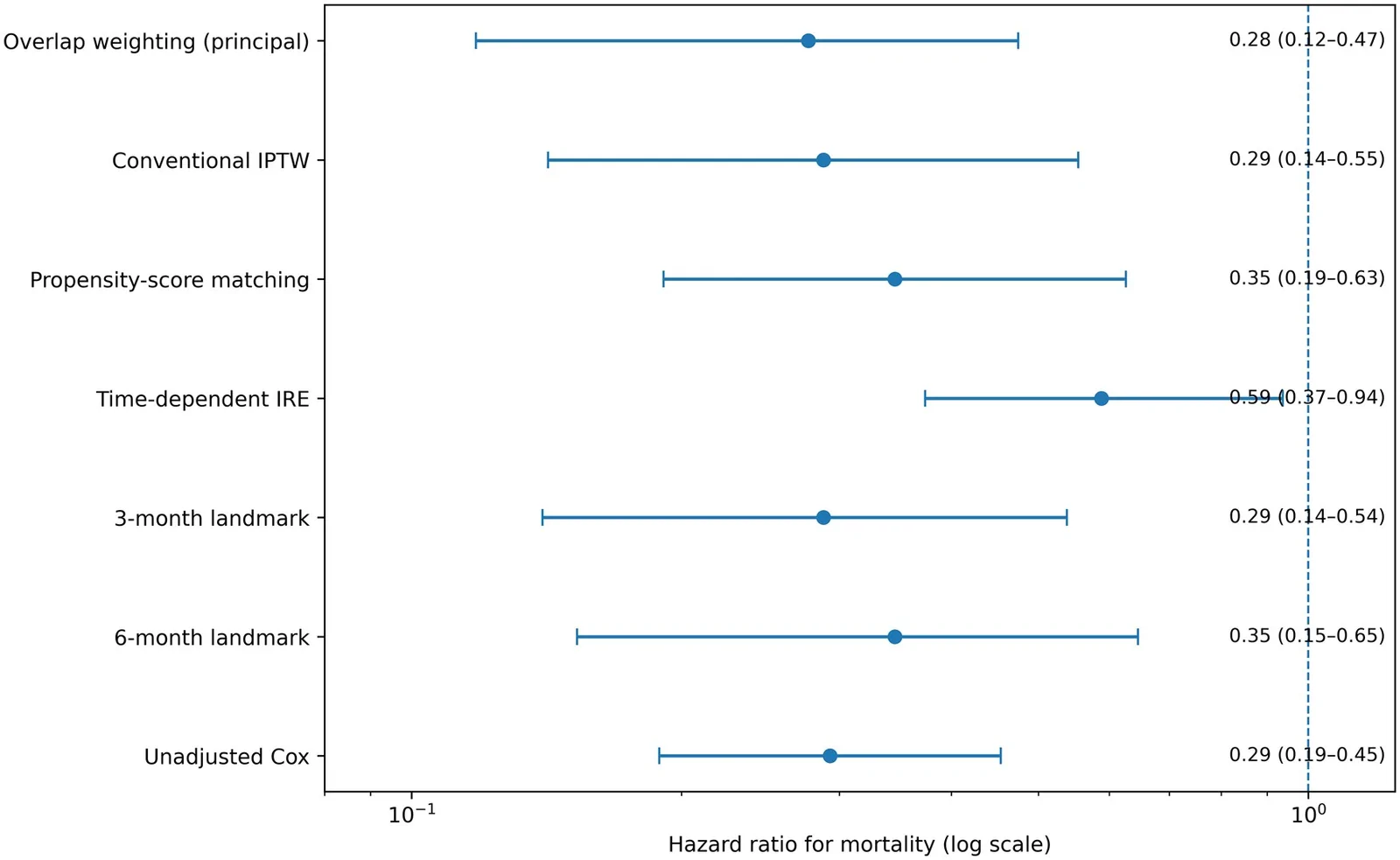
Association between an IRE-based treatment strategy and overall survival across primary and sensitivity analyses. Forest plot displaying hazard ratios (HRs) and 95% CIs for mortality comparing patients treated within an irreversible electroporation (IRE)-based treatment strategy with those receiving standard of care (SOC) without IRE. The principal adjusted observational analysis used propensity-score overlap weighting and demonstrated an HR of 0.28 (95% bootstrap CI, 0.12-0.48). The direction of association was consistent in sensitivity analyses using conventional inverse probability of treatment weighting (IPTW; HR, 0.29; 95% bootstrap CI, 0.14-0.55), propensity-score matching (HR, 0.35; 95% CI, 0.19-0.63), the 3-month landmark (HR, 0.29; 95% bootstrap CI, 0.14-0.54), and the 6-month landmark (HR, 0.35; 95% bootstrap CI, 0.15-0.65). In the time-dependent Cox model, in which patients contributed unexposed person-time until the actual date of IRE treatment, the magnitude of the association was attenuated but remained present (HR, 0.59; 95% bootstrap CI, 0.37-0.94). This analysis was performed specifically to reduce immortal-time bias related to the interval between diagnosis and receipt of IRE. The unadjusted Cox estimate (HR, 0.29; 95% CI, 0.19-0.45) is shown for descriptive comparison. Estimates below 1.0 favor the IRE-based treatment strategy. IRE, irreversible electroporation; SOC, standard of care; IPTW, inverse probability of treatment weighting; HR, hazard ratio.

Of the 12 IRE patients who were treated percutaneously, 4 patients had expired at the time of this analysis, with a mean survival of 12 months. Eight percutaneous patients were still alive, with a mean follow-up of 25 months.

Median PFS reported in Table 3, in months, was 9 (95% CI: 7-12) for the IRE cohort overall, and 6 (95% CI: 5-8) for SOC. The hazard ratio comparing PFS of the IRE cohort to the SOC cohort was 0.6 (95% CI: 0.4-0.9; *P* < .05).

**Table 3.** Progression-free survival stage III safety population.

Analysis type	Characteristics	Treatment received	
		IRE (*N* = 99)	SOC (*N* = 38)
**Primary analysis**	Median survival in months (95% CI)	9 (7-12)	6 (5-8)
	Log-rank test *P*-value	<.01	
**Sensitivity analysis**	Hazard ratio (95% CI)	0.6 (0.4-1)	
	Cox model *P*-value	<.05	
**Disease status**	Censored at date of last follow-up	14 (14%)	2 (5%)
	Death	27 (27%)	10 (26%)
	Disease progression	58 (59%)	26 (68%)
**Type of progression**	Distant	39 (67%)	12 (46%)
	Local	15 (26%)	12 (46%)
	Regional	2 (3%)	2 (8%)
	Unknown	2 (3%)	0 (0%)
**Location of metastases^a^**	Bone	1 (3%)	1 (8%)
	Liver	15 (39%)	6 (50%)
	Lung	6 (15%)	2 (17%)
	Peritoneum	11 (28%)	5 (42%)
	Other	6 (15%)	1 (8%)

Progression‑free survival outcomes and progression characteristics in the stage III safety population by treatment group.

a More than one location is possible for a given patient, so percentages may not sum to 100%.

### Safety

Overall, 80% (79/99) of IRE patients and 95% (36/38) of SOC patients experienced an AE after enrollment. The timing of AEs from enrollment is shown in Table S2. For the IRE cohort, 29% (29/99) of patients had IRE-related AE with abdominal pain (8%, 8/99), fatigue (5%, 5/99), nausea (3% 3/99), pancreatic fistula (3%, 3/99), and vomiting (3%, 3/99) being the most common. Corresponding proportions in the SOC cohort were 36/38 (95%) and 27/38 (71%) for any adverse event and grade ≥3 events, respectively. A larger proportion of the SOC cohort had chemotherapy-related AEs than the IRE cohort: 68% (26/38) vs 22% (22/99).

Total, 56% (55/99) of the IRE cohort and 71% (27/38) of the SOC cohort had a Grade 3 or greater AE. The most common Grade 3 or greater AEs for the IRE cohort were anemia (11%, 11/99), sepsis (8%, 8/99), and gastrointestinal hemorrhage (7%, 7/99), and for the SOC cohort were abdominal pain (13%, 5/38), ascites (13%, 5/38), anemia (11%, 4/38), sepsis (11%, 4/38), hypokalemia (11%, 4/38), and diarrhea (11%, 4/38) (Table S4).

## Discussion

Pancreatic cancer is frequently diagnosed when resection is not feasible, leaving systemic therapy with or without local treatment as the principal therapeutic strategy.^1–3^ In the DIRECT registry, patients treated within an IRE-based treatment strategy had longer observed survival than patients receiving SOC without IRE. Importantly, the magnitude of this association depended on the analytic approach. The unadjusted HR from diagnosis was approximately 0.29, whereas a time-dependent analysis that classified patients as unexposed until the actual date of IRE produced a more conservative HR of 0.59. This attenuation is clinically and methodologically important because it demonstrates that differential treatment timing and the period during which patients must remain alive and progression-free before IRE account for part, but not all, of the observed survival difference.

The principal adjusted analysis used propensity-score overlap weighting because conventional IPTW did not achieve adequate balance across all measured baseline covariates. Overlap weighting substantially improved covariate balance and yielded an adjusted HR of 0.28 (95% bootstrap CI, 0.12-0.48). The corresponding 24-month RMST difference was 4.99 months (95% bootstrap CI, 1.15-8.12), providing an absolute measure of the observed survival association. These estimates should not be interpreted as proving an independent causal effect of IRE; rather, they demonstrate that the survival association persisted after emphasizing patients with the greatest measured clinical overlap between treatment strategies.

The findings were directionally consistent across alternative analytic approaches, including conventional IPTW, propensity-score matching, and 3- and 6-month landmark analyses. The matched analysis, which included 29 IRE-SOC pairs, yielded an HR of 0.35, and the 6-month landmark analysis yielded an HR of 0.35. The E-value for the overlap-weighted point estimate was 6.68 and for the confidence-limit closest to the null was 3.63, suggesting that a substantial unmeasured association would be required to fully explain the adjusted estimate. Nevertheless, the *E*-value cannot exclude residual confounding and should be interpreted only as a quantitative sensitivity analysis.

In the current study, a lower proportion of patients in the IRE cohort had AEs overall compared to the SOC cohort (80%, 79/99 vs 95%, 36/38). The IRE cohort also had a lower incidence of AEs that were Grade 3 + (56%, 55/99 vs 71%, 27/38) suggesting that IRE has a safety profile similar to SOC. Cross-cohort safety comparisons should be interpreted cautiously because adverse-event exposure windows and treatment pathways differed between groups. However, the percentage of patients with a Grade 3+ AE was higher than that of studies reported in a systematic review of IRE which ranged from 1% to 42%. While this range is considerable, the reasons for this variation may be due to differences in treatment protocols, size of treated tumors, physician experience with the IRE procedure, or method of delivery (open vs percutaneous).^21^ Additionally, this prospective IDE was rigorously designed to collect AE data and therefore may have been more sensitive to the capture of AEs compared to smaller retrospective studies. Regarding the high rate of AEs in the SOC cohort, patients with pancreatic cancer tend to experience AEs due to the severity of their illness especially as the disease progresses post-treatment. As an example, the percentage of patients with a Grade 3+ AE in the SOC cohort (71%, 27/38) was comparable to the rate of grade 3 or 4 AEs among patients randomized to modified FOLFIRINOX (76%) in an RCT randomizing patients to adjuvant chemotherapy.^22^

The DIRECT Registry study was not designed to compare IRE with radiation or preoperative radiation; rather, it was designed to highlight a potential role for IRE as an adjunct or alternative in selected cases, as optimal local therapy for patients with unresectable disease or margins-at-risk remains an unresolved question.^23^^,^^24^ Future randomized studies and multidisciplinary decision-making would be essential to define the optional sequencing and selection of local therapies. The imbalance in sample size between the SOC and IRE cohorts reflects real-world treatment allocation and differential eligibility for IRE. More importantly, the treatment groups demonstrated limited overlap in several measured clinical characteristics. Conventional IPTW improved but did not fully eliminate this imbalance; therefore, overlap weighting was selected as the principal adjusted analysis because it focuses inference on patients with the greatest probability of receiving either treatment strategy. This approach improves measured covariate comparability but does not address unmeasured differences in disease biology, referral patterns, multidisciplinary selection, or physician treatment preference.

This study had several limitations. The study experienced difficulty with enrollment into the SOC cohort as some patients specifically sought IRE treatment. An additional limitation is that the registry did not achieve the sample size specified in the original study protocol. The protocol planned enrollment of 532 patients, whereas the final comparative cohort included 137 patients. This discrepancy resulted from patient bias and patient desire for IRE treatment, rather than an outcome-driven decision to restrict the analytic population. The smaller-than-planned cohort reduces statistical precision, particularly for subgroup analyses, and should be considered when interpreting the magnitude of the observed survival associations.

There is also heterogeneity in the treatment pathways represented by both cohorts. SOC after induction therapy for Stage III PDAC is not uniform, and chemotherapy and radiation exposures varied between patients. In addition, the IRE-based cohort included patients treated with tumor ablation and patients undergoing margin accentuation in association with pancreatic resection. Because the subject-level dataset used for the present propensity and survival reanalysis did not contain a patient-level resection designation, the contribution of pancreatic resection could not be separated from the broader IRE-based treatment strategy in the adjusted analyses. Finally, although the time-dependent Cox analysis substantially reduces immortal-time bias by assigning IRE exposure only after the procedure occurs, residual selection bias and unmeasured confounding remain unavoidable in this nonrandomized registry.

## Conclusion

In this multicenter observational registry, treatment within an IRE-based strategy was associated with longer overall survival compared with SOC without IRE after adjustment for measured baseline differences. The association persisted across overlap weighting, IPTW, propensity-score matching, landmark analyses, and a time-dependent treatment model, although the more conservative time-dependent estimate was attenuated relative to the unadjusted comparison. These findings support further prospective comparative evaluation but should not be interpreted as establishing an independent causal survival effect of IRE.

## Supplementary Material

oyag416_Supplementary_Data

## Data Availability

The datasets presented in this article are not readily available because the data are part of an ongoing study. Data are planned to be shared upon the completion of the study and its submission to the FDA. The data that supports the findings of this study will be available from the corresponding author upon reasonable request.
