# Supplementary material for: Association of irreversible electroporation treatment with overall survival in Stage III pancreatic ductal adenocarcinoma: DIRECT registry results
Source: Oncologist. 2026 Oct 14;31(11):oyag416. doi: 10.1093/oncolo/oyag416 (PMC13722508; doi:10.1093/oncolo/oyag416)
Supplement: Supplementary file 1 [file oyag416_supplementary_data.zip › Supplementary Methods v2.docx]

**Supplementary Methods. Study eligibility criteria for the DIRECT Registry.** Patient inclusion and exclusion criteria.

**Inclusion Criteria**

All the following criteria were met in order to be enrolled and continue participation in the study:

1. Provision of signed and dated informed consent form

2. Patient was 18 years of age and older

3. Patient had a diagnosis of Stage 3 pancreatic (PC) cytologically or pathologically confirmed per American Joint Committee on Cancer (AJCC) staging criteria

4. Patient had a tumor evaluated as Stage 3 according to National Comprehensive Cancer Network (NCCN) guidelines, based on radiographic imaging or exploratory surgery

5. Maximum axial and anterior to posterior tumor dimension of ≤3.5 cm after standard of care (SOC)

6. Patient received three months of SOC per each participating institution’s guidelines

7. Patient had an Eastern Cooperative Oncology Group (ECOG) performance status of 0 or 1

8. Patient had an American Society of Anesthesiologists (ASA) classification of physical health

status of 1, 2, 3 or 4

9. Patients at IRE sites who were deemed eligible for IRE received ablation using the NanoKnife System

10. Patient showed no evidence of disease progression based on NCCN guidelines after completing three months of SOC

**Exclusion Criteria**

A potential patient was excluded from the study if he/she met any of the following exclusion criteria:

1. Participation in an interventional trial for PC during the study data collection period

2. Pregnant or lactating patients or male or female patients of reproductive potential who were not willing to employ highly effective birth control from screening to 6 months after the last dose of chemotherapy

3. Patients who were unable to tolerate general anesthetic with full skeletal muscle blockade

4. Patients with the presence of implanted cardiac pacemakers, defibrillators, electronic devices or implanted devices with metal parts in the thoracic cavity at the time of IRE

Supplemental Methods:

The site enrollment periods were as follows: University of Louisville (10/04/2021 to 27/09/2024), Northwest Community Healthcare (15/08/2019 to 27/09/2023), University of South Florida and Tampa General Hospital (20/12/2019 to 07/12/2022), Baptist Hospital of Miami and Miami Cancer Institute (22/11/2019) to (27/09/2023), University of California San Diego (17/04/2020 to 27/09/2024), Atrium Health (24/02/2020 to 16/12/2022), Columbia University (09/06/2020 to 27/09/2023), UT Southwestern Medical Center (30/05/2020 to 02/12/2022), Barnes-Jewish Hospital and Washington University School of Medicine (08/01/2020 to 01/12/2022), Mayo Clinic Minnesota (05/03/2021 to 27/09/2023), Mayo Clinic Florida 04/02/2021 to 01/12/2022), WellStar Summit Surgical (30/03/2020 to 02/12/2022), Capital Health System, Inc (12/05/2020 to 01/12/2022), St. Luke’s University Health Network (02/06/2020 to 01/12/2022), University of Iowa (21/10/2020 to 17/06/2024), Augusta University (03/12/2020 to 25/05/2022), University of Florida Gainesville (15/05/2021 to 07/12/2022), and NYU Langone Health (26/03/2021 to 21/08/2023).

Eligibility:

Males and Females, 18 years of age or older with pathologically confirmed American Joint Committee on Cancer (AJCC) stage 3 PDAC with Eastern Cooperative Oncology Group (ECOG) performance status of 0 or 1 and no evidence of disease progression based on National Comprehensive Cancer Network (NCCN) guidelines after completing three months of SOC chemotherapy were eligible for inclusion in the study. Exclusion criteria included participation in an interventional trial for pancreatic cancer during the data collection period and inability to tolerate general anesthetic with full skeletal muscle blockade.

Study Procedures:

The Prior to study initiation, all participating physicians at IRE sites were trained on optimal tissue specific use procedures for the NanoKnife System, including procedure settings and post-procedure follow-up. Sites were requested to adhere to guidelines for the treatment and monitoring of patients, testing markers (Carbohydrate Antigen 19-9 [CA 19-9]) and performing imaging (computed tomography [CT] or magnetic resonance imaging [MRI]) at least every three to four months during the first two years

.

The study received approval from the Western Institutional Review Board (IRB approval 2019-0965) and local IRBs. All prospective patients provided written informed consent. The retrospective cohort was also prospectively consented for data collection. The study was conducted in accordance with the International Conference on Harmonization’s Good Clinical Practice guidelines.

Assessments and Data Collection

Data elements were entered into electronic case report forms at baseline, at the index hospitalization for IRE patients, and at 3-month intervals until study exit (death, withdrawal, loss to follow-up) or a minimum of 24 months of follow up was obtained. At each evaluation from the time of enrollment, sites were instructed to determine whether an adverse event (AE) occurred including relationship to underlying disease, procedure, and/or treatments. AEs were graded based on Common Terminology Criteria for Adverse Events (CTCAE) grade 1 (mild) to 5 (death).

Endpoints:

An independent Clinical Endpoint Adjudication Committee (CEC) was established during this study. The main purpose was to review and adjudicate individual patient AEs and serious AEs (SAEs). The CEC consisted of three members with expertise in pancreatic cancer management. None of the members had direct involvement in the conduct of the study or had any conflicts of interest. A Data Monitoring Committee was also established to review cumulative study data to evaluate safety, study conduct, and scientific validity and integrity of the trial. The Data Monitoring Committee (DMC) consisted of three physicians and a statistician who were independent from the trial and had no conflicts of interest.

Statistical Analysis:

Descriptive analyses. Baseline demographic, disease, and treatment characteristics were summarized by treatment group using mean and standard deviation or median and interquartile range for continuous variables and frequency and percentage for categorical variables. Between-group balance was assessed primarily using absolute standardized mean differences (SMDs), rather than significance testing alone. An absolute SMD <0.10 was considered evidence of adequate covariate balance.

Primary estimand and time origin. The primary effectiveness analysis evaluated OS from diagnosis. The principal adjusted estimand was the association between assignment to an IRE-based treatment strategy and mortality among patients within the region of clinical equipoise represented by the overlapping propensity-score distributions. Survival from study enrollment was retained as a secondary descriptive analysis. Diagnosis was selected as the primary time origin because time from diagnosis to registry enrollment differed substantially between treatment groups and an analysis beginning solely at enrollment could be affected by differential patient selection and treatment timing.

Propensity-score modeling. A propensity score for receipt of an IRE-based treatment strategy was estimated using multivariable logistic regression. Clinically prespecified baseline covariates included age, sex, ECOG performance status, baseline tumor dimensions, baseline CA19-9, induction chemotherapy regimen, duration or number of cycles of induction chemotherapy, receipt of pre-enrollment radiation therapy, year of diagnosis, and participating center where data completeness and sample size permitted. Variables were selected on clinical grounds rather than univariable statistical significance.

Overlap weighting and IPTW. Propensity-score overlap weights were used for the principal adjusted observational analysis because conventional stabilized inverse probability of treatment weighting (IPTW) improved but did not fully eliminate covariate imbalance in this dataset. For overlap weighting, treated patients were weighted by 1 minus the propensity score and SOC patients by the propensity score, thereby emphasizing patients with the greatest probability of receiving either treatment strategy and reducing the influence of observations with limited common support. Covariate balance before and after weighting was assessed using absolute standardized mean differences (SMDs), with an absolute SMD <0.10 considered evidence of adequate balance. Weighted Cox proportional-hazards models and weighted survival curves were used to estimate the association between treatment strategy and OS. Conventional stabilized IPTW was retained as a prespecified sensitivity analysis, with propensity-score and weight distributions examined for non-overlap and extreme weights.

Restricted mean survival time. RMST was used as a complementary absolute measure of survival association and was calculated as the area under the weighted survival curve through 24 months from diagnosis. The absolute difference in 24-month RMST between treatment groups was reported with bootstrap 95% confidence intervals. RMST was emphasized because it does not require the proportional-hazards assumption and provides an interpretable estimate of the difference in average survival time accumulated during the prespecified follow-up interval.

Landmark analyses. To examine the potential influence of differential survival and treatment-selection periods after diagnosis, prespecified landmark analyses were conducted among patients alive and without documented disease progression at 3 months and 6 months after diagnosis. Follow-up for each landmark analysis began at the respective landmark date, and only patients satisfying the landmark eligibility criteria were included. Kaplan-Meier estimates, RMST differences, and Cox models were generated from each landmark. These analyses were considered sensitivity analyses rather than substitutes for the primary analysis because IRE treatment may occur after the landmark time.

Time-dependent treatment analysis. Because receipt of IRE occurred after diagnosis and the time between diagnosis and IRE varied among patients, an additional Cox model treated IRE exposure as a time-dependent covariate. Patients contributed person-time to the non-IRE state from diagnosis until the date of IRE and to the IRE-exposed state thereafter. This analysis was performed specifically to reduce immortal-time bias that could arise if patients were classified as IRE-treated from the date of diagnosis despite receiving the procedure later in their disease course.

Propensity-score matched sensitivity analysis. As a secondary assessment of robustness to the weighting approach, 1:1 nearest-neighbor propensity-score matching without replacement was performed using a caliper of 0.2 standard deviations of the logit of the propensity score. This yielded 29 matched IRE-SOC pairs. Covariate balance was assessed using standardized mean differences, and survival was analyzed in the matched population. Because matching reduced the available sample size, this analysis was considered supportive rather than the principal adjusted analysis. The number of bootstrap replicates; whether resampling occurred at the patient or center level; whether the propensity model/weights were re-estimated within each replicate; and percentile vs BCa confidence intervals.

Treatment-strategy and resection sensitivity analyses. Because a subset of patients in the IRE cohort underwent pancreatic resection in conjunction with IRE, the independent effect of IRE cannot be inferred from a pooled treatment-strategy comparison. A patient-level resection/margin-accentuation indicator was not available in the subject-level dataset used for the present propensity and survival reanalysis; therefore, a definitive IRE-without-resection versus SOC analysis could not be performed from this dataset alone. The influence of resection is addressed as an important limitation, and a resection-restricted sensitivity analysis should be completed when the patient-level procedure designation is available.

Sensitivity analyses. Robustness of the principal overlap-weighted analysis was evaluated using: (1) unadjusted Cox regression; (2) conventional stabilized IPTW; (3) propensity-score matching; (4) 3- and 6-month landmark analyses; and (5) a time-dependent Cox model in which patients contributed unexposed person-time from diagnosis until the actual date of IRE and exposed person-time thereafter. The time-dependent analysis was specifically intended to reduce immortal-time bias. Concordance in the direction and approximate magnitude of estimates across analyses was emphasized rather than statistical significance from any individual model. Because overlap weighting changes the target population, add the weighted effective sample size (ESS) overall and preferably by treatment group. This tells readers how much information remains after weighting.

Unmeasured confounding. An E-value was calculated for the principal overlap-weighted hazard ratio and for the confidence-limit closest to the null. The E-value quantifies the minimum strength of association that an unmeasured confounder would need to have with both treatment assignment and mortality, conditional on the measured covariates, to fully explain the observed association. This analysis was interpreted as a sensitivity assessment and not as evidence that residual confounding was absent.

Progression and competing risks. PFS was defined as time from the specified origin to documented disease progression or death from any cause, whichever occurred first, and was evaluated using survival methods analogous to those used for OS. Because death is included within the composite PFS endpoint, death was not treated as a competing event for PFS. For analyses of specific progression outcomes, including local progression and distant metastatic progression, death without the progression event of interest was treated as a competing event. Cumulative incidence functions were used to estimate event incidence, with Fine-Gray sub distribution hazard models used for adjusted comparisons where event numbers were sufficient.

Proportional-hazards assessment. The proportional-hazards assumption for Cox models was evaluated using Schoenfeld residuals and graphical assessment of log-minus-log survival plots. If clinically meaningful nonproportionality was identified, time-varying coefficients and RMST were preferentially reported rather than relying solely on a single Cox hazard ratio.

Missing data. The extent and pattern of missing baseline covariate data were reported for each treatment group. If missingness in covariates required for adjustment was nontrivial, multiple imputation by chained equations was used under a missing-at-random assumption, with the outcome indicator and survival time incorporated into the imputation model. Estimates across imputed datasets were combined using Rubin's rules. Complete-case analyses were performed as a sensitivity analysis. Outcome events were not imputed.
