# Supplementary material for: Association of irreversible electroporation treatment with overall survival in Stage III pancreatic ductal adenocarcinoma: DIRECT registry results
Source: Oncologist. 2026 Oct 14;31(11):oyag416. doi: 10.1093/oncolo/oyag416 (PMC13722508; doi:10.1093/oncolo/oyag416)
Supplement: Supplementary file 1 [file oyag416_supplementary_data.zip › Suppl Table 4.docx]

**Supplemental Table 4 . Grade 3+ adverse events experienced by the patient population.**

|  | | Treatment received | |
| --- | --- | --- | --- |
| Body System | Dictionary Term | IRE (N=99) | SOC (N=38) |
| Overall |  | 55 (56%) | 27 (71%) |
| Blood And Lymphatic System Disorders |  | 11 (11%) | 6 (16%) |
|  | Anaemia | 11 (11%) | 4 (11%) |
|  | Leukocytosis | 1 (1%) | 0 (0%) |
|  | Neutropenia | 1 (1%) | 2 (5%) |
|  | Pancytopenia | 0 (0%) | 1 (3%) |
|  | Thrombocytopenia | 1 (1%) | 0 (0%) |
| Gastrointestinal Disorders |  | 27 (27%) | 19 (50%) |
|  | Abdominal Distension | 1 (1%) | 0 (0%) |
|  | Abdominal Pain | 6 (6%) | 5 (13%) |
|  | Abdominal Pain Upper | 0 (0%) | 1 (3%) |
|  | Ascites | 4 (4%) | 5 (13%) |
|  | Colitis | 3 (3%) | 0 (0%) |
|  | Constipation | 0 (0%) | 1 (3%) |
|  | Diarrhoea | 0 (0%) | 4 (11%) |
|  | Duodenal Obstruction | 1 (1%) | 0 (0%) |
|  | Gastrointestinal Haemorrhage | 7 (7%) | 2 (5%) |
|  | Gingival Erythema | 0 (0%) | 1 (3%) |
|  | Haematemesis | 1 (1%) | 0 (0%) |
|  | Intestinal Perforation | 1 (1%) | 0 (0%) |
|  | Intra-Abdominal Haemorrhage | 2 (2%) | 0 (0%) |
|  | Melaena | 1 (1%) | 0 (0%) |
|  | Nausea | 1 (1%) | 3 (8%) |
|  | Obstruction Gastric | 1 (1%) | 2 (5%) |
|  | Pancreatic Failure | 0 (0%) | 1 (3%) |
|  | Pancreatitis | 2 (2%) | 0 (0%) |
|  | Pancreatitis Acute | 1 (1%) | 0 (0%) |
|  | Pneumatosis Intestinalis | 1 (1%) | 0 (0%) |
|  | Small Intestinal Obstruction | 2 (2%) | 0 (0%) |
|  | Upper Gastrointestinal Haemorrhage | 0 (0%) | 2 (5%) |
|  | Vomiting | 4 (4%) | 2 (5%) |
| General Disorders and Administration Site Conditions |  | 17 (17%) | 8 (21%) |
|  | Adverse Drug Reaction | 0 (0%) | 1 (3%) |
|  | Asthenia | 2 (2%) | 0 (0%) |
|  | Chest Pain | 1 (1%) | 0 (0%) |
|  | Death | 1 (1%) | 0 (0%) |
|  | Disease Progression | 5 (5%) | 0 (0%) |
|  | Fatigue | 3 (3%) | 2 (5%) |
|  | Hypothermia | 1 (1%) | 0 (0%) |
|  | Malaise | 0 (0%) | 2 (5%) |
|  | Pain | 4 (4%) | 1 (3%) |
|  | Pyrexia | 4 (4%) | 2 (5%) |
|  | Ulcer Haemorrhage | 0 (0%) | 1 (3%) |
| Hepatobiliary Disorders |  | 10 (10%) | 1 (3%) |
|  | Bile Duct Obstruction | 1 (1%) | 0 (0%) |
|  | Bile Duct Stenosis | 3 (3%) | 0 (0%) |
|  | Biliary Tract Disorder | 1 (1%) | 0 (0%) |
|  | Cholangitis | 2 (2%) | 0 (0%) |
|  | Cholecystitis | 3 (3%) | 0 (0%) |
|  | Cholecystitis Acute | 1 (1%) | 0 (0%) |
|  | Hyperbilirubinaemia | 0 (0%) | 1 (3%) |
|  | Jaundice | 1 (1%) | 0 (0%) |
|  | Portal Vein Stenosis | 1 (1%) | 0 (0%) |
|  | Portal Vein Thrombosis | 1 (1%) | 0 (0%) |
| Infections and Infestations |  | 24 (24%) | 11 (29%) |
|  | Abdominal Abscess | 3 (3%) | 0 (0%) |
|  | Abdominal Infection | 2 (2%) | 0 (0%) |
|  | Appendicitis | 1 (1%) | 0 (0%) |
|  | Arthritis Bacterial | 0 (0%) | 1 (3%) |
|  | Bacteraemia | 2 (2%) | 2 (5%) |
|  | Biliary Tract Infection Bacterial | 1 (1%) | 0 (0%) |
|  | Cellulitis | 0 (0%) | 1 (3%) |
|  | Clostridiodes Difficile Sepsis | 1 (1%) | 0 (0%) |
|  | Clostridium Difficile Infection | 1 (1%) | 0 (0%) |
|  | Corona Virus Infection | 1 (1%) | 0 (0%) |
|  | Emphysematous Cholecystitis | 0 (0%) | 1 (3%) |
|  | Gastroenteritis | 1 (1%) | 0 (0%) |
|  | Hepatic Infection | 1 (1%) | 0 (0%) |
|  | Infection | 2 (2%) | 0 (0%) |
|  | Klebsiella Bacteraemia | 0 (0%) | 1 (3%) |
|  | Liver Abscess | 1 (1%) | 1 (3%) |
|  | Pancreatic Abscess | 1 (1%) | 0 (0%) |
|  | Pneumonia | 3 (3%) | 0 (0%) |
|  | Postoperative Wound Infection | 1 (1%) | 0 (0%) |
|  | Retroperitoneal Abscess | 1 (1%) | 0 (0%) |
|  | Sepsis | 8 (8%) | 4 (11%) |
|  | Septic Shock | 5 (5%) | 1 (3%) |
|  | Skin Infection | 1 (1%) | 0 (0%) |
|  | Urinary Tract Infection | 0 (0%) | 1 (3%) |
|  | Vascular Access Site Infection | 1 (1%) | 0 (0%) |
|  | Wound Infection | 1 (1%) | 0 (0%) |
| Nervous System Disorders |  | 3 (3%) | 4 (11%) |
|  | Cerebrovascular Accident | 1 (1%) | 0 (0%) |
|  | Encephalopathy | 1 (1%) | 1 (3%) |
|  | Neuropathy Peripheral | 0 (0%) | 1 (3%) |
|  | Syncope | 1 (1%) | 1 (3%) |
|  | Toxic Encephalopathy | 0 (0%) | 1 (3%) |

Grade 3 or higher adverse events observed across body systems in patients treated with IRE or SOC.

IRE = irreversible electroporation; SOC = standard of care.

^a^Includes adverse events with body system prevalence >10% for at least one of the study cohorts.
