# Supplementary material for: Association of irreversible electroporation treatment with overall survival in Stage III pancreatic ductal adenocarcinoma: DIRECT registry results
Source: Oncologist. 2026 Oct 14;31(11):oyag416. doi: 10.1093/oncolo/oyag416 (PMC13722508; doi:10.1093/oncolo/oyag416)
Supplement: Supplementary file 1 [file oyag416_supplementary_data.zip › Supplemental Table 1.docx]

**STable 1**. **Procedure details for the IRE cohort.**

|  | | IRE |
| --- | --- | --- |
|  | | (N=99) |
| Treatment Intent | Margin accentuation | 37 (37%) |
|  | Tumor ablation | 62 (63%) |
| Major Adjunctive Procedures |  | 70 (71%) |
|  | Cholecystectomy | 29 (29%) |
|  | Distal Pancreatectomy | 5 (5%) |
|  | Gastrojejunostomy | 6 (6%) |
|  | Hepaticojejunostomy | 8 (8%) |
|  | J-tube | 25 (25%) |
|  | Lymphadenectomy | 13 (13%) |
|  | Portal Vein or SMV Resection | 4 (4%) |
|  | Splenectomy | 8 (8%) |
|  | Subtotal Pancreatectomy | 3 (3%) |
|  | Subtotal Pancreatectomy with Celiac Resection | 2 (2%) |
|  | Whipple | 25 (25%) |
|  | Other | 20 (20%) |
| Number of Days from Procedure to Discharge | Mean (S.D.) Median (Q1 - Q3) | 7 (5) 6 (4 - 8) |
| Total Procedure Time (Minutes) | n Mean (S.D.) Median (Q1 - Q3) | 91 335 (171) 295 (210 - 428) |
| Number of Probes | 2 | 25 (25%) |
|  | 3 | 15 (15%) |
|  | 4 | 24 (24%) |
|  | 5 | 2 (2%) |
|  | 6 | 1 (1%) |
|  | Multiple | 4 (4%) |
|  | Unknown | 28 (28%) |
| Probe Configuration | Diamond | 10 (10%) |
|  | Line | 25 (25%) |
|  | Square | 8 (8%) |
|  | Three Triangles | 3 (3%) |
|  | Triangle | 19 (19%) |
|  | Multiple | 4 (4%) |
|  | Unknown | 30 (30%) |
| Number of Pulses Delivered | n Mean (S.D.) Median (Q1 - Q3) | 70 1007 (876) 655 (300 - 1570) |
| Pulse Length (usec) | 70 | 1 (1%) |
|  | 70-90 | 8 (8%) |
|  | 80-90 | 3 (3%) |
|  | 90 | 59 (60%) |
|  | Unknown | 28 (28%) |
| Total IRE Delivery Time (Minutes) | n Mean (S.D.) Median (Q1 - Q3) | 71 53 (54) 36 (11 - 74) |

Summary of key procedural characteristics, device parameters, and adjunctive surgical interventions for the IRE cohort.

IRE = irreversible electroporation

Note: NanoKnife device data was not available for every patient in the population. Therefore, the n for each data point in this table will differ from the overall N of the analysis population.
