# Supplementary material for: Association of irreversible electroporation treatment with overall survival in Stage III pancreatic ductal adenocarcinoma: DIRECT registry results
Source: Oncologist. 2026 Oct 14;31(11):oyag416. doi: 10.1093/oncolo/oyag416 (PMC13722508; doi:10.1093/oncolo/oyag416)
Supplement: Supplementary file 1 [file oyag416_supplementary_data.zip › Supplemental Table 2.docx]

**STable 2. Adverse events by enrollment timepoint.**

|  | Treatment received Number (%) of Patients | |  |  |
| --- | --- | --- | --- | --- |
| Timepoint | IRE (N=99) | SOC (N=38) | P-Value |  |
| Overall | 79 (80%) | 36 (95%) | 0.03 |  |
| 0-7 Days | 27 (27%) | 9 (24%) |  |  |
| 8-30 Days | 48 (49%) | 19 (50%) |  |  |
| 31-90 Days | 46 (47%) | 26 (68%) |  |  |
| 91-180 Days | 34 (34%) | 19 (50%) |  |  |
| >180 Days | 44 (44%) | 22 (58%) | 0.7 |  |
| Adverse events reported by timepoints from enrollment for patients undergoing IRE treatment versus SOC.  IRE = irreversible electroporation; SOC = standard of care.  Note: Timepoint is measured by days from enrollment. | | | | |
