# Supplementary material for: Association of irreversible electroporation treatment with overall survival in Stage III pancreatic ductal adenocarcinoma: DIRECT registry results
Source: Oncologist. 2026 Oct 14;31(11):oyag416. doi: 10.1093/oncolo/oyag416 (PMC13722508; doi:10.1093/oncolo/oyag416)
Supplement: Supplementary file 1 [file oyag416_supplementary_data.zip › Supplemental Table 3.docx]

Supplemental Table 3: Total Treatments for IRE Treated Group and SOC Treated Group

| Treatment characteristic | IRE (n=99) | SOC (n=38) |
| --- | --- | --- |
| Any pre-enrollment FOLFIRINOX-containing regimen | 73 (74%) | 24 (63%) |
| Any pre-enrollment gemcitabine/nab-paclitaxel-containing regimen | 37 (37%) | 13 (34%) |
| Pre-enrollment chemotherapy cycles, median (IQR) | 3 (1–8) | 4 (4–7) |
| Patients with available pre-enrollment cycle counts | 85 | 37 |
| Pre-enrollment radiation | 38 (38%) | 1 (3%) |
| Second-line chemotherapy after enrollment | 36 (36%) | 28 (74%) |
| Second-line cycles among recipients, median (IQR), as reported | 4 (3–7) | 5 (4–8) |
| Cumulative chemotherapy cycles, first through third lines, median (IQR) | 6 (2–12) | 11 (7–13) |
| Post-enrollment radiation | 29 (29%) | 15 (40%) |
| Radiation at any time | 64 (65%) | 16 (42%) |
